# Study on the Tribological Properties of Multilayer Concentric Hexagonal Laser Texturing on Rubber Surfaces of Screw Pumps

**DOI:** 10.3390/ma17153708

**Published:** 2024-07-26

**Authors:** Xinfu Liu, Xinglong Niu, Chunhua Liu, Xiangzhi Shi, Yi Sun, Zhongxian Hao, Shouzhi Huang, Yuan Wang, Hua Tao

**Affiliations:** 1 Key Lab of Industrial Fluid Energy Conservation and Pollution Control (Ministry of Education), Qingdao University of Technology, Qingdao 266520, China; 2 College of Mechanical and Electronic Engineering, China University of Petroleum (East China), Qingdao 266580, China; 3 Research Institute of Exploration & Development, PetroChina, Beijing 100083, China; 4 Lusheng Petroleum Development Co., Ltd., China Petroleum & Chemical Corporation, Dongying 257077, China

**Keywords:** stator rubber, multilayer concentric arrangement, hexagonal texture, reduce friction and drag, fluid lubrication

## Abstract

Given the friction and drag reduction effects observed in various biological hexagonal structures in nature, a new design was implemented on the rubber surface of the stator of a submersible screw pump. This design featured a multilayer concentric hexagonal groove structure. Furthermore, a composite multilayer hexagonal structure integrating grooves and pits was also developed and applied. This study investigated the influence of groove layer number, groove depth, pit depth, and multilayer hexagonal groove texture arrangement on the rubber surface flow characteristics. Additionally, the pressure field state, the degree of influence on the oil film-bearing capacity, and the biomimetic and hydrodynamic lubrication theories were tested using the finite element analysis method. Tribological experiments were conducted on nanosecond laser-processed rubber textures under simulated liquid lubrication conditions, reflecting actual shale oil well experiments. These experiments aimed to investigate the influence of multilayer hexagonal shape parameters on the tribological characteristics of the stator-rotor friction pair of a submersible screw pump. The results indicated that with a constant overall size, a multilayer hexagonal structure with ~0.1 mm groove depth enhanced the oil film-bearing capacity, providing significant friction and drag reduction. For composite textures, a deeper pit depth within the study area enhanced the oil film-bearing capacity. Furthermore, a gradient arrangement of groove textures featuring wider outer grooves and shallower depth exhibited superior performance in terms of bearing capacity.

## 1. Introduction

Submersible screw pumps are rodless artificial lifting devices that combine the advantages of both volumetric and centrifugal pumps [1,2,3,4,5,6]. They are widely employed in onshore oil recovery wells with high sand content and high gas-to-liquid ratios [7], as well as offshore oil platforms [8,9]. In the oil recovery process, the stator of the electric submersible screw pump is highly susceptible to wear due to stator-rotor friction, leading to screw pump failure. The rubber friction wear of the stator in electric submersible screw pumps is a significant factor in reducing the lifespan of these pumps. This issue is exacerbated under complex downhole conditions and the influence of lifting liquids. Researchers across various fields have conducted extensive studies on the friction wear of the rubber stator in screw pumps [10,11,12,13,14,15,16,17]. They aim to improve the tribological performance between the stator and rotor, enhancing the service life of the pump and achieving energy savings and consumption reduction.

In reality, there are no perfectly smooth, frictionless surfaces. However, the natural unevenness of biological surfaces often aids in lubrication and friction reduction. For instance, the scales on the skin of sharks significantly reduce resistance during swimming [18]. Inspired by nature, the development of bionic friction pairs prepared in regularly arranged geometric shapes has been proven to be an effective way to enhance the tribological performance of friction pairs [19,20,21,22,23,24,25,26,27,28].

For the study of the surface texture shape, the researchers made a lot of investigations using computational fluid dynamics (CFD) simulations and experiments [29,30,31,32,33]. Zhao et al. [34] discussed the friction reduction mechanisms and theoretical models under different lubrication conditions and reviewed the geometrical characteristics of surface texturing and recent progress in improving the tribological properties of material surfaces under practical working conditions. Zhang et al. proposed the preparation of a surface texture on the rubber surface of a stator to improve its tribological characteristics to reduce friction, prolong the service life, and improve the working efficiency [35]. Two main categories of surface texture patterns have been studied: pits and grooves [36]. Research indicates that groove-shaped textures are particularly effective in enhancing hydrodynamic pressure [37,38]. As research progressed, the study of surface textures has increasingly focused on complex structures within these textures [39,40,41,42].

**Figure 1:**
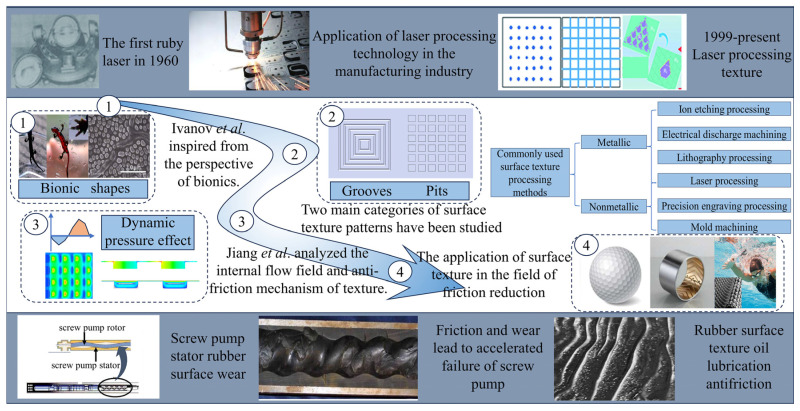
Research status of surface texture [40,60].

In 1960, Theodore Meyman invented the first ruby laser. Laser processing technology has been extensively researched and applied in various industrial fields [43,44,45,46]. Furthermore, compared with other surface machining technologies, laser processing offers high precision, fast rate, low environmental pollution, and affordability. In fields of tool surfaces [47], gears [48], plain bearings [49], cylinder liners [50], pistons [50], and mechanical seals [51], laser-machined surface textiles are often employed with lubricating fluids to reduce friction [52,53,54]. The tribological properties of these textures are significantly influenced by different operating conditions. Moreover, the quality of the lase-processed texture is significantly affected by the laser processing power parameters. For instance, Andersson et al. found that suitable laser-textured surfaces can reduce friction on steel surfaces [55]. Wu et al. obtained the appropriate laser energy and scanning rate to make the processed surface structure clear and complete [56]. Mao et al. found that scanning power and scanning speed affect the processing depth of surface texture. Roushan [57] demonstrated that using an appropriate high-energy laser beam and scanning frequency to prepare linear textures on the surface of a cemented friction pair could reduce wear during movement [58,59](Figure 1). Therefore, the selection of appropriate laser processing parameters is crucial for enhancing the quality of rubber surface textures. Finally, this study aimed to explore the influence of multilayer concentric hexagonal groove structure shape parameters on the rubber surface pressure field and flow state. Additionally, it investigated the optimal laser power processing parameter through experimental research to address the wear issues of rubber surfaces in electric submersible screw pump stators.

## 2. Modeling the Stator Internal Surface Texture of a Progressive Cavity Pump Using a Tribological Experimental Method

### 2.1. Simulation Model of a Multilayer Concentric Hexagonal Texture Structure

A hexagonal structure with multiple concentric grooves was designed and constructed using the bionic-derived single-layer hexagonal grooves as the base. Additionally, the hexagonal pit factor was introduced to construct a multilayer composite hexagonal braid with hexagonal grooves and pits. The number of hexagonal grooves, the groove depth, and the pit depth were controlled to study their influence on the tribological properties of the hexagonal braid. The multilayer hexagonal groove structure was deformed to experimentally explore the effect of two gradient arrangements of unequal grooves on the performance of the hexagonal structure, considering three layers of equal-sized grooves. The effects of concentric grooves of unequal width and depth on the lubrication properties of multilayer concentric hexagonal textures were investigated. Overall, the dynamic pressure lubrication effect and friction reduction performance of multilayer hexagonal groove structures were enhanced.

**Figure 2:**
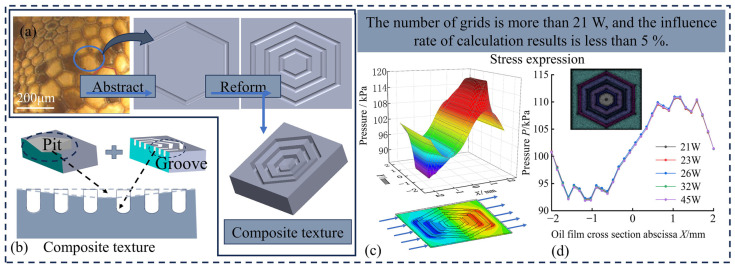
(**a**–**d**) Hexagonal texture simulation model.

A fluid simulation model of a multilayer concentric hexagonal groove structure was established. Shale oil fluid medium with a density of 870 kg/m^3^ and a viscosity of 0.08 mPa·s, was set to flow into the model from the *x*-axis in the positive direction at a speed of 1 m/s (Figure 2c). The inlet and outlet pressures were set to 101 kPa.

A three-dimensional fluid model was established using the CFD finite element calculation and fluid analysis software (Workbench Fluent 2020). The average mesh quality was 6.21 (Figure 2d). When the mesh exceeded 200,000 elements, the influence rate of the increased mesh number on the calculation was <5%.

### 2.2. Mathematical Models of Fluid Dynamic Pressure Theory

The following assumptions were made:

(1) Volumetric forces, such as gravity and magnetism, were not considered.
(2) There was no relative sliding of the fluid surface at the solid interface.
(3) Pressure variation in the direction of film thickness was not considered, and the fluid at the surface of the friction pair was a Newtonian fluid of constant viscosity.
(4) Translational speed was considered instead of moving speed without accounting for the curvature of the oil film.
(5) The fluid on the surface of the friction pair experienced laminar flow.
(6) In comparison with viscous drag, inertial forces, including the force of fluid acceleration and the force of oil film curvature, were neglected.
(7) The viscosity along the direction of the lubricant film was constant.

The Reynolds equation for the fluid on the weaving surface was obtained under these assumptions:

(1) $$\frac{\partial }{\partial x}\left(\frac{\rho h^{3}}{\eta }\frac{\partial p}{\partial x}\right)+\frac{\partial }{\partial y}\left(\frac{\rho h^{3}}{\eta }\frac{\partial p}{\partial y}\right)=6\left[\frac{\partial }{\partial x}\left(U\;\rho h\right)+\frac{\partial }{\partial x}(V\rho h)+2\rho (w_{h}-w_{0})\right]$$

where *η* is the fluid viscosity (Pa·s), and *p* is the lubricating film pressure in N. Furthermore, *U* and *V* are the relative velocities of the friction pair along the x and y directions, respectively. *w*_h_ and *w*_0_ are the velocities of the two surfaces of the friction pair along the direction of the lubricating film thickness.

Furthermore, assuming that the viscosity was constant in the gap and disregarding the effects of temperature and pressure on the viscosity, the Reynolds equation for a locally pressurized fluid film was derived (Equation (2)):

(2) $$\frac{\partial }{\partial x}\left(\frac{\rho h^{3}}{\eta }\frac{\partial p}{\partial x}\right)+\frac{\partial }{\partial y}\left(\frac{\rho h^{3}}{\eta }\frac{\partial p}{\partial y}\right)=6U\frac{\partial }{\partial x}\left(\rho h\right)$$

When considering only the dynamic pressure effect, the Reynolds equation was simplified to Equation (3). This simplified Reynolds equation was adopted in the theoretical model of the weaving structure under a small gap, where an incompressible fluid dominated the laminar lubrication state (Equation (3)):

(3) $$\frac{\partial }{\partial x}\left(h^{3}\frac{\partial p}{\partial x}\right)+\frac{\partial }{\partial y}\left(h^{3}\frac{\partial p}{\partial y}\right)=6U\eta \frac{\partial h}{\partial x}$$

The standard K-epsilon turbulence model [38] is as follows:

(4) $$\frac{\partial }{\partial t}\left(\rho k\right)+\frac{\partial }{\partial x_{i}}\left(\rho ku_{i}\right)=\frac{\partial }{\partial x_{i}}\left[\left(\mu +\frac{\mu_{t}}{\sigma_{k}}\right)\frac{\partial k}{\partial x_{i}}\right]+G_{k}+G_{b}-\rho_{\varepsilon }-Y_{M}+S_{k}$$

(5) $$\frac{\partial }{\partial t}\left(\rho \varepsilon \right)+\frac{\partial }{\partial x_{j}}\left(\rho \varepsilon u_{i}\right)=\frac{\partial }{\partial x_{j}}\left[\left(\mu +\frac{\mu_{t}}{\sigma_{\varepsilon }}\right)\frac{\partial \varepsilon }{\partial x_{j}}\right]+C_{1\varepsilon }\frac{\varepsilon }{k}\left(G_{k}+C_{3\varepsilon }G_{b}\right)-C_{2\varepsilon }\rho \frac{\varepsilon^{2}}{k}+S_{\varepsilon }$$

where the constants are set as follows: (i) 1.44 for the $C_{1\varepsilon }$; (ii) 1.92 for the $C_{2\varepsilon }$; (iii) 1.0 for the $\sigma_{k}$; and (iv) 1.3 for the $\sigma_{k}$.

Additionally, by integrating the pressure of the lubricant film, the bearing capacity of the lubricant film was obtained, and the expression for bearing capacity was derived as follows:

(6) W=∬pdxdy

The shear stress on the surface of the friction pairs was integrated, and the magnitude of the resistance was obtained.

(7) F=∬τdxdy

Given the resistance and load-bearing force, the friction factor of the friction pair was obtained through their ratio, with the expression provided as follows:

(8) $$\mu =\frac{F}{W}$$

### 2.3. Experimental Method for Tribological Properties of Textured Rubber

**Figure 3:**
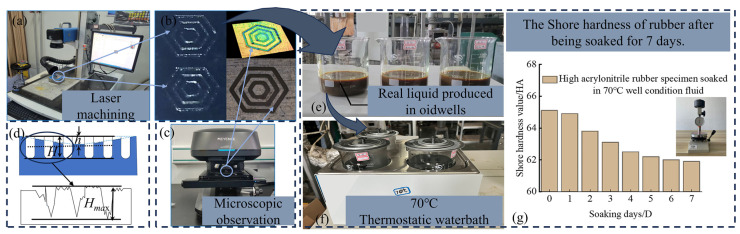
(**a**–**g**) Actual well condition and 70 °C immersion condition.

A specific type of high-acrylonitrile rubber was selected as the experimental material. The structure of the rubber surface was textured and simulated under oil extraction conditions (Figure 3a). The rubber samples were soaked in the actual extraction fluid from shale oil wells at 70 °C for 7 days. Subsequently, friction experiments were conducted under lubrication conditions with the actual extraction fluid. The stator rubber was textured using an IPG nanosecond laser, and the textured rubber material was observed using a three-dimensional (Figure 3c) confocal microscope (VK-X1050), Japan KEYENCE (Osaka, Japan).

As shown in Figure 3d, *H* is the depth of the groove designed in the simulation, *h* is the depth of the pit designed in the simulation, and *H_max_* is the maximum depth of the laser-machined grooves.

The nanosecond laser processing of rubber specimens was characterized by its simplicity and effective modeling, meeting the requirements for experimental processing. To simulate underground oil production conditions, the rubber specimen was soaked in the actual production liquid at 70 °C for 7 days (Figure 3e,f), ensuring the experimental results correlated with the actual application conditions. The variation of the average hardness values of the high-acrylonitrile rubber specimens utilized in the experiments is shown in Figure 3g. The hardness exhibited a decreasing trend, stabilizing after 7 days of immersion in the actual well-conditioned fluid at 70 °C, decreasing from 65.1 HA initially to 61.9 HA.

**Figure 4:**
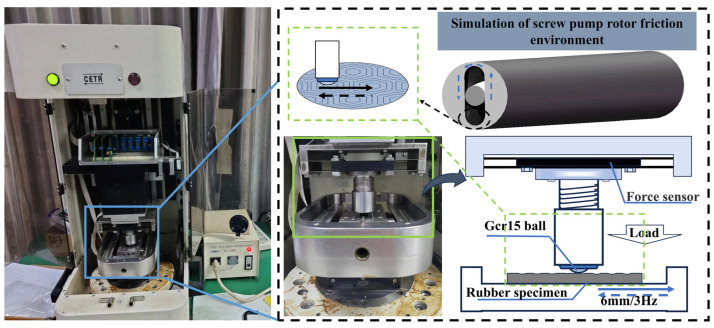
Experimental conditions of UMT friction testing machine.

Tribological tests were conducted on screw pump stator rubber with a textured surface after 7 days of immersion using a UMT-3 reciprocating friction and wear tester, U.S. CETR products, with the textured screw pump stator rubber specimen placed in the lower part and a 25 mm diameter Gar 15 bearing steel ball in the upper part of the specimen. The two specimens underwent relative sliding at a fixed frequency of 3 Hz (reciprocating distance of 6 mm). Friction experiments were conducted at a constant speed, with a load of 10 N applied under lubrication conditions to simulate actual well conditions in shale oil wells for 1200 s (Figure 4).

## 3. Fluid Simulation Characterization of Woven Surfaces 

### 3.1. Effect of Inflow Direction on the Properties of Multilayer Hexagonal Textures

**Figure 5:**
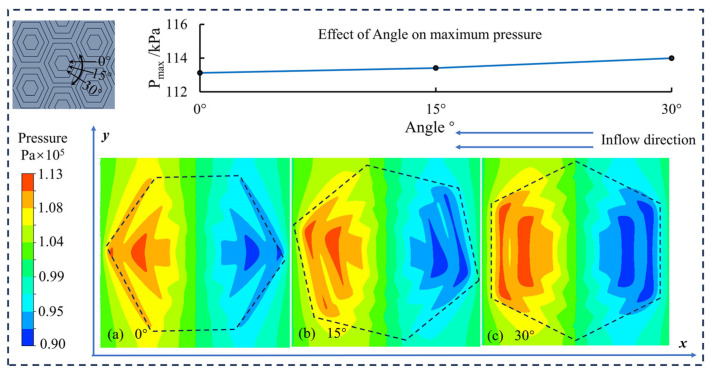
Influence of inflow direction ((**a**) 0°, (**b**) 15°, (**c**) 30°) on the dynamic pressure effect of multilayer hexagonal weaves.

Figure 5 depicts different inflow angles for the variation of pressure in the fluid domain of the weave, using a three-layer hexagonal groove weave as an example. The hexagonal shape has good symmetry, so it can be simplified to the effect of angle on the dynamic pressure effect of the weave in the range of 0° to 30°. Among them, 30° inflow direction has the best pressure performance, the maximum pressure on the upper wall is larger compared to other angles, and the oil film has a better carrying capacity.

Therefore, in the subsequent sections of this paper, the inflow direction of the fluid for the CFD simulation and the friction direction for the friction test are explored in the direction of 30°.

### 3.2. Effect of Groove Depth on the Properties of Multilayer Hexagonal Textures

**Figure 6:**
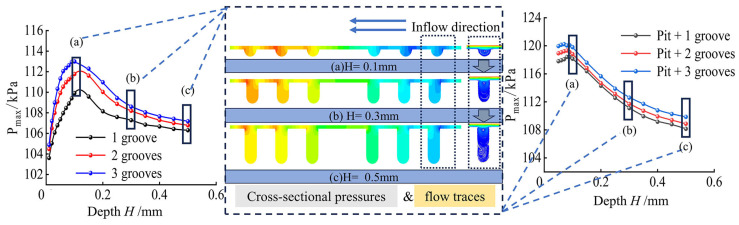
Pressure cloud map of trench section in different layers.

The graphs in Figure 6 show the relationship between maximum pressure and groove depth on the upper wall of the multilayer concentric hexagonal texture, varying with different numbers of turns. The maximum pressure experienced a rapid increase from 0.01 mm to 0.09 mm deep, peaking at 0.1 mm dep. Subsequently, it decreased and plateaued at 0.3 mm deep.

Meanwhile, Figure 6 shows the pressure situation of the hexagonal groove texture with a different number of layers in the cross-section of the groove, along with a change in the flow field within the groove, corresponding to the change of the groove depth. Pressure variations were presented by a sequential lightening of pressure colors from the outer to the inner laps. Darker colors signify areas of higher pressure, indicating a larger additional net-bearing capacity and superior oil film-bearing capacity. In the three-layer groove hexagonal structure, the outer two layers of grooves predominantly enhanced pressure. The change in the cross-section of the texture oil film included variations in fluid dynamic pressure during the flow process. Despite the axisymmetric texture positive and negative pressure areas being roughly equal in area, the wedge-shaped cross-section amplified the effect of fluid dynamic pressure. Consequently, the deviation from the standard pressure value was more pronounced in the high-pressure zone compared with the low-pressure zone, thereby enhancing the overall carrying capacity. This differential pressure contributed to an additional net load-bearing capacity across the entire oil film, consequently affecting the performance of the structure. A higher oil film-bearing capacity enhanced the tribological performance of the structure, leading to friction and drag reduction.

The increases in groove depth initially enhanced the pressurizing effect of the outermost groove, expanding its high-pressure area beyond that of the inner two cycles of the grooves. However, as the groove depth increased, the high-pressure area within the groove occupied a smaller proportion of the entire groove. Consequently, the pressurizing effect of the groove became weak.

The groove flow line exhibited profound changes in fluid flow state with increasing groove depth. For instance, between a groove depth of 0.01 mm and 0.1 mm, the groove formed a vortex, resulting in complete formation beyond a depth of 0.1 mm. The changes in oil film cross-section due to enhanced dynamic pressure mitigated energy loss induced by vortex formation, consequently increasing surface pressure on the oil film. Subsequently, an increase in groove depth led to further expansion of the vortex, accompanied by an increase in energy loss, ultimately resulting in a decrease in maximum pressure on the film surface. However, when the groove depth exceeded 0.3 mm, the influence of the bottom fluid on the upper fluid diminished and pressure at the groove tended to stabilize. At a groove depth of ~0.5 mm, a new vortex was formed, which rapidly increased in volume.

Conclusively, increasing the groove depth altered the fluid flow state in the groove, leading to vortex formation and energy loss in the oil film flow. The cross-section of the pressure curve indicated a decline in maximum oil film pressure at a groove depth of >0.1 mm, thereby reducing the oil film-bearing capacity and tribological performance of the texture.

### 3.3. Effect of Pit Depth on the Properties of Composite Multilayer Hexagonal Texture

**Figure 7:**
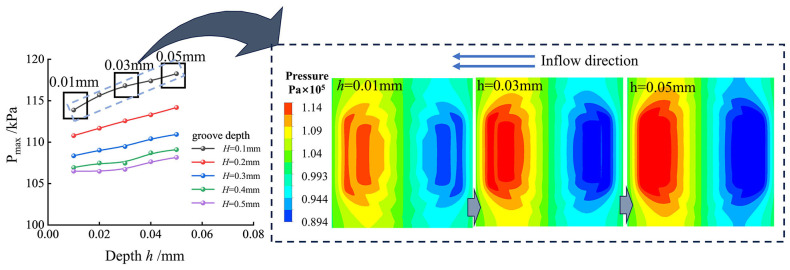
Variation of oil film pressure with pit depth.

The addition of pit elements, equivalent in size to the outermost grooves, into the concentric hexagonal grooves of the multilayers enhances the pressure-bearing capacity of the texture to a certain extent. The depth of these pits resulted in different performances of the pressure-bearing capacity of the texture. Figure 7 shows the variation in the oil film pressure with pit depth for a hexagonal texture with pits and multilayer grooves. The presence of pits increased the oil film pressure across the texture, and the maximum oil film pressure of the composite hexagonal grooved texture increased with increasing pit depth.

The effect of pit depth on the performance of the composite hexagonal texture was investigated under the conditions of constant groove depth (Figure 7). The maximum pressure of the fluid on the surface of the texture increased with increasing pit depth. An increase in pit depth from 0.01 mm to 0.05 mm gradually enlarged the high-pressure area formed by the grooves on the surface until the divided high-pressure region was connected. This facilitated the more complete development of dynamic pressure effects due to cross-sectional changes, consequently enhancing the load-carrying capacity of the oil film.

Meanwhile, the maximum pressure of the composite hexagonal texture of pits and multilayer grooves correlated with the change of the multilayer hexagonal texture concerning groove depth. Furthermore, with groove depths exceeding 0.1 mm, the maximum pressure of oil film tended to decrease with increasing groove depth. Thus, the rate of decrease diminished gradually.

### 3.4. Analysis of Multilayer Weaving Flow Field with Trench Gradient Arrangement

**Figure 8:**
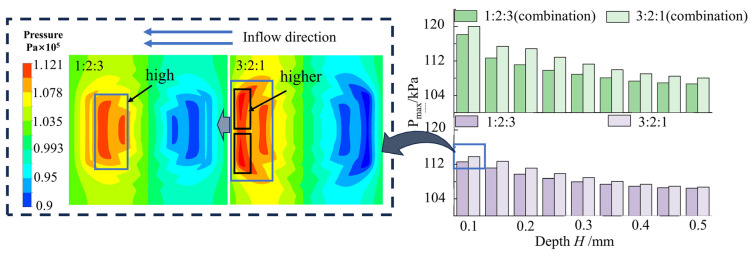
Variation of pressure of oil film with gradient texture of different widths and groove depth.

Figure 8 shows the maximum pressure of a multilayer hexagonal groove texture with different width gradients with increasing groove depth. The three-layer groove widths were represented as 1:2:3 and 3:2:1, respectively. The maximum pressure on the upper surface of the oil film for the multilayer hexagonal groove weave with a width gradient of 1:2:3 from outer to inner was lower compared with a width gradient of 3:2:1. Both sets of width gradient groove weaves exhibited similar pressure characteristics to the conventional multilayer hexagonal groove weave. As the depth of the grooves increased from 0.1 mm to 0.5 mm, the pressure gradually decreased and then plateaued.

The pressure distribution shown in Figure 8 indicates the arrangement of the 1:2:3 and 3:2:1 groove width at a groove depth of 0.1 mm. In these configurations, high pressure tends to concentrate in the two wilder grooves. The wilder the outermost circles of the grooves, the higher the maximum pressure value. A wider groove width enhanced the dynamic effect on the groove cross-section, leading to more effective development and a greater likelihood of achieving higher maximum pressure. In the multilayer grooves analyzed above, the primary pressure-bearing role was assumed by the outer two circles of grooves, necessitating wider outer grooves in the groove arrangement and facilitating increased bearing capacity of the structure.

**Figure 9:**
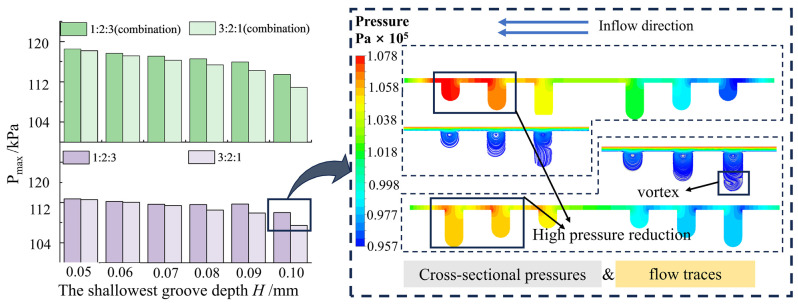
Variation of oil film pressure with grooves depth in different gradients.

Figure 9 illustrates the variation of wall pressure on the oil film for two sets of multilayered hexagonal groove weaves with varying groove depth gradient rows as the depth of the shallowest groove increased sequentially.

A multilayer hexagonal groove texture with a 1:2:3 groove depth gradient from outer to inner exhibited higher maximum pressure on the upper wall of the oil film and superior load-bearing capacity of the oil film compared with a multilayer hexagonal groove texture of 3:2:1 groove depth gradient. Maximum pressure decreased with increasing depth.

The pressure maps at the cross-sections of the grooves elucidated the above phenomenon. The pressure map of the groove cross-section, combined with the description of the pressure map at the cross-section of the multilayer hexagonal texture by the depth of the grooves in Section 3.1 above, indicated that the outer two grooves primarily bear pressure in the multilayer hexagonal texture with three turns of grooves. In the 1:2:3 groove depth arrangement, the pressure was greater in the outermost and middle groove sections, with minimal energy loss in the deepest grooves in the absence of vortices. Conversely, in the 3:2:1 groove depth gradient arrangement, the outermost grooves were affected by the energy loss due to the formation and development of vortices. Thus, it was difficult for hydrodynamic effects to counteract the energy loss from the vortices, resulting in lower maximal pressure on the oil film.

A comparison of two hexagonal textures with two different groove widths and depth gradients revealed that multilayer groove textures with wider outermost groove widths and shallower depths, as well as groove gradient rows, exhibited exceptional performance.

## 4. Rubber Friction Experiment on Fluid Texturing in Real Well Conditions

**Figure 10:**
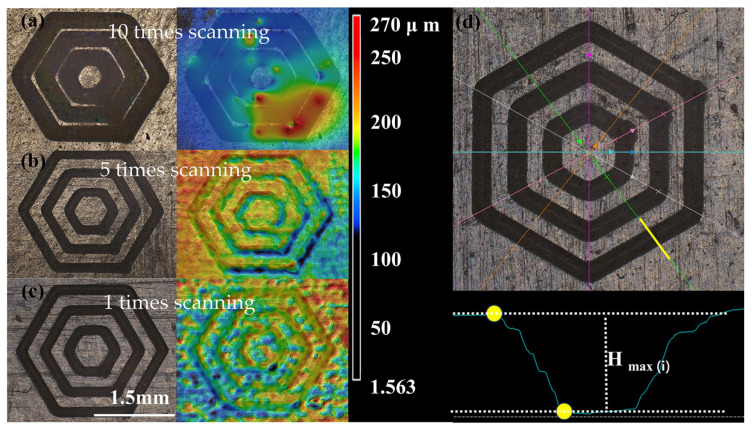
Influence of laser processing parameters on the morphology of rubber textures (**a**–**c**) and measurement of groove depth (**d**).

The selection of appropriate laser processing parameters was crucial for enhancing the quality of surface textile processing. The surface morphology of the rubber texture was processed using a nanosecond laser with (a) ten times scanning (Figure 10a), (b) five times scanning (Figure 10b), (c) one times scanning (Figure 10c). Notably, at a constant laser power of 20 W, the surface texture of the rubber sample exhibited superior processing quality with <5 scans.

The study used the Keyence three-dimensional confocal microscope (VK-X1050). Japan KEYENCE, to observe and analyze the depth of the grooves of the multilayer hexagonal weave. The study took two auxiliary sections in each of the six directions of the weave and measured the perpendicular distance between the highest point and the lowest point of the groove at the maximum depth of the grooves here, *H_max_*(*i*). The average value was the maximum depth of the multilayer hexagonal groove weave, *Hmax*.

Multilayer hexagonal textures with depths of 0.01 mm, 0.05 mm, 0.1 mm, and 0.15 mm were selected for nanosecond laser processing and tribological experiments. Table 1 gives the depths of the rubber weave grooves after nanosecond laser processing as observed and analyzed with Keyence three-dimensional confocal microscope (VK-X1050), Japan KEYENCE.

Standard deviation was calculated as follows:

(9) $$\sigma =\sqrt{\frac{\sum_{i=1}^{12}{\left(H_{max}(i)-\bar{H_{\max}}\right)}^{2}}{12}}$$

**Figure 11:**
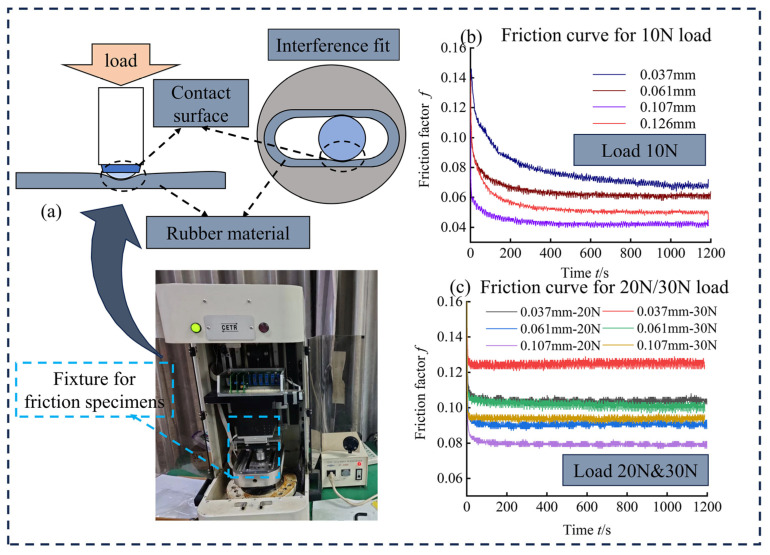
(**a**–**c**) Friction test of textured rubber specimen after soaking.

The experiment was conducted under actual well conditions, employing liquid lubrication in an oilfield with shale oil. This setup simulated the working environment of a downhole screw pump for tribological experiments. Applying different loads to better simulate the friction characteristics of rubber after pressure deformation during downhole screw pump stator-rotor interference fit (Figure 11a). The friction coefficient of the rubber specimen exhibited a decreasing trend during the first 200 s of the friction experiment due to the high initial pressure. Subsequently, it gradually reached a plateau after 200 s. The friction factor value after the stabilization of the friction curve served as the standard for evaluating the tribological performance of different textures.

Figure 11b shows the tribological characteristic curves of four nanosecond laser-processed multilayer hexagonal texture types with different groove depths under a 10 N load condition. Among these, the smallest friction factor was observed for the multilayer hexagonal texture with a groove depth of 0.107 mm, followed by the multilayer hexagonal texture with a groove depth of 0.126 mm. Conversely, the highest friction factor value was observed in the multilayer hexagonal texture with a groove depth of 0.037 mm. This indicated that the friction and drag reduction effects of multilayer grooves were initially enhanced and subsequently weakened within ~0.1 mm groove depth. Thus, with the groove depth range of ~0.1 mm, the friction and drag reduction effects of multilayer grooved hexagonal textures exhibited a clear relationship; deeper groove depths of 0.1 mm resulted in an enhanced effect.

As a type of positive displacement pump, the fit between the stator and rotor of the screw pump is typically an interference fit. To achieve better sealing and thereby obtain higher volumetric efficiency, the UMT reciprocating tester, which applies load caused by deformation, can simulate very well the rubber deformation of the stator-rotor interference fit of the screw pump. Different applied loads result in different deformations of the rubber, which leads to different friction coefficients of the rubber. For example, we changed the applied load to 20 N and 30 and found that the higher the load, the higher the coefficient of friction. The reason for this phenomenon is that, as the rubber weave grooves receive pressure extrusion, the amount of lubricant stored inside the grooves decreases, which affects the dynamic pressure lubrication effect and causes the coefficient of friction to increase in the presence of increased loads.

**Figure 12:**
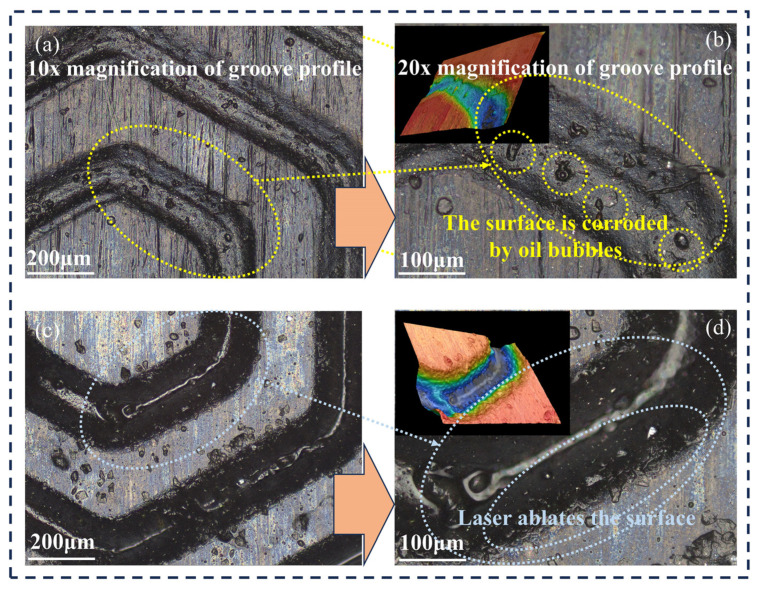
(**a**–**d**) Microscopic image of laser-machined rubber surface after oil immersion(10× & 20×).

Figure 12 gives the surface image of the laser-machined texturized rubber after an experiment with actual well-fluid lubricated friction under 30 N load conditions. Laser ablation marks on the groove edges are evident in Figure 12d. Tiny corrosion pits are more likely to appear in the laser heat radiation area inside and around the edges of the grooves in Figure 12b after oil soaking. The rubber used in the experiment is a special wear-resistant rubber for the stator bushing of the screw pump. The presence of the weaving structure also enhances the pressure-bearing capacity of the rubber surface. There are no obvious wear traces after the 1200 s friction experiment.

## 5. Conclusions

(1) The stator rubber surface of the screw pump was designed with woven textures. Simulations of oil film fluid on the textured surface and tribological characteristics under actual well lubrication conditions indicated that a suitable multilayer hexagonal groove texture enhanced the bearing capacity of the surface, exhibiting a more effective friction and drag reduction.
(2) In simulation studies and tribological experiments, it was observed that increasing the depth of hexagonal grooves to ~0.1 mm significantly enhanced the load-carrying capacity as well as friction and drag reduction. However, the increase in groove depth at ≥0.1 mm led to larger vortex volume inside the grooves, forming new vortexes and resulting in a greater energy loss and a decrease in load-carrying capacity.
(3) For composite multilayer hexagonal textures combining pits and grooves, the depth of the pits significantly affected the load-carrying capacity. The study revealed that deeper pits led to higher maximum pressures on the oil film, thereby increasing the load-carrying capacity.
(4) Among the four types of multilayer grooved textures with unequal groove arrangements, a quantitative gradient arrangement method was employed. Fluid simulation analysis indicated that the outermost groove was wider in width and shallower in depth compared with the inner groove, exhibiting superior load-carrying capacity and a more effective friction and drag reduction.
(5) Through the friction experiments of weaving structure oil lubrication under different loads, this study found that the larger the normal load suffered by the rubber specimen in the study range, the larger the coefficient of friction, which corresponded to the stator rotor of the screw pump being more likely to cause serious wear with more interference fit.

## Figures and Tables

**Table 1:** Machining depth in nanoseconds.

Number	Design Depth (mm)	Number of Scans	Scanning Power (W)	Maximum Machining Depth (mm)	SD
1	0.01	2	20	0.037	0.00947
2	0.05	2	20	0.061	0.00723
3	0.1	5	20	0.107	0.00682
4	0.15	5	20	0.126	0.00578

## Data Availability

The raw data supporting the conclusions of this article will be made available by the authors on request.
